# Nocturnal enuresis: prevalence and risk factors among school-aged children with sickle-cell anaemia in a South-east Nigerian city

**DOI:** 10.1186/s13052-015-0176-9

**Published:** 2015-09-29

**Authors:** Chizoma I. Eneh, Henrietta U. Okafor, Anthony N. Ikefuna, Samuel N. Uwaezuoke

**Affiliations:** Enugu State University Teaching Hospital (ESUTH), Enugu, Nigeria; Department of Paediatrics, University of Nigeria Teaching Hospital (UNTH), Ituku-Ozalla, Enugu, Nigeria

**Keywords:** Sickle-cell anaemia, Nocturnal enuresis, Prevalence, Risk factors, School-aged children

## Abstract

**Background:**

Sickle-cell anaemia (SCA) is the most common inherited haemoglobinopathy affecting the Negroid race. Renal complications such as enuresis can occur during childhood. Reports show that children and adolescents with SCA may be at a higher risk of nocturnal enuresis than their counterparts with normal haemoglobin genotype.

**Aims:**

The study aims to determine the prevalence of nocturnal enuresis and possible risk factors among school-aged children with SCA in a South-east Nigerian city.

**Methods:**

A hospital-based and cross-sectional descriptive study of 70 school-aged children with SCA who met the study criteria, and 70 age- and sex-matched controls with normal haemoglobin genotype was conducted in the Paediatric Sickle-cell Anaemia Clinic of the University of Nigeria Teaching Hospital (UNTH) Enugu. Data was subjected to multivariate analysis using logistic regression model with nocturnal enuresis as the dependent variable and the possible risk factors as the independent variables.

**Results:**

The prevalence of nocturnal enuresis among the Subjects and the Controls was 31.4 and 21.4 % respectively (*p* = 0.180). It was significantly higher among male Subjects (48.7 %) than among male Controls (23.1 %) [OR (95 % CI) =8.14 (2.12–31.24), *p* < 0.001]; and among Subjects whose parents had a childhood history of enuresis [OR (95 %) =10.39 (2.45–44.05), *p* = 0.002]. The difference in the prevalence of enuresis in the female cohort was however not significant.

**Conclusions:**

Children with SCA have a tendency to develop nocturnal enuresis when compared to their non-affected counterparts. Male gender and parental childhood history of nocturnal enuresis are potential socio-demographic risk factors.

## Introduction

Sickle-cell anaemia (SCA) is the most common inherited haemoglobinopathy which affects millions of people in the Tropics especially sub-Saharan Africa [1], and is also common among African-Americans [2]. Nigeria accounts for the largest number of cases worldwide with a prevalence of about 20 per 1000 births [3].

Infarction-related renal complications start from childhood [4], and include tubulopathies like impaired ability to concentrate urine (manifesting as hyposthenuria, polyuria and enuresis) and abnormalities of urine acidification [5–7].

The Diagnostic and Statistical Manual of Mental Disorders-IV (DSM-IV) criterion defines enuresis as the repeated voiding of urine into bed or clothes which may be involuntary or unintentional, the behaviour being clinically significant if manifested by either a frequency of voiding twice weekly for at least 3 consecutive months or the presence of significant distress or impairment in social, academic (occupational) or other important areas of functioning by a child five years or older and the behaviour not being due exclusively to the direct physiological effect of a substance (a diuretic) or a general medical condition (e.g., diabetes, spina bifida, seizure disorder) [8].

Several studies in both developed and developing countries which evaluated the prevalence of enuresis among children and adolescents with SCA show that they may be at a higher risk of nocturnal enuresis than their counterparts with normal haemoglobin genotype [9–14]. For instance, Akinyanju et al. [12] reported a high prevalence value of 41.6 % in South-west Nigeria among paediatric SCA patients compared to 18.5 % in their counterparts with normal haemoglobin. Among children with SCA in Congo Brazzaville, other researchers [9] documented a prevalence value of 50.9 and 16.4 % among their peers with normal haemoglobin genotype. However, there appears to be dearth of data on the prevalence of enuresis among affected children in the south-eastern part of Nigeria. Furthermore, abnormalities of urinalysis such as proteinuria, leucocyturia and haematuria have also been noted in other studies conducted in South-eastern part of the country [15, 16].

The present study was thus conducted to determine the prevalence of and the risk factors for nocturnal enuresis among school-aged children with SCA seen in a tertiary health facility in Enugu, South-east Nigeria.

## Subjects and methods

### Study site/design/population

The study was conducted at the University of Nigeria Teaching Hospital (UNTH) - a major tertiary health facility located within Enugu metropolis, South-east Nigeria. It was a descriptive, cross-sectional study in which 70 children with SCA and their 70 age- and sex-matched controls were recruited consecutively. The Subjects were drawn from school-aged children attending the weekly Paediatric Sickle Cell Clinic of the hospital while the Controls were their classmates with normal haemoglobin genotype.

### Study criteria/sampling method

After obtaining ethical approval from the Health Research and Ethics Committee of the hospital and written informed consent from the parents/caregivers, the patients were enrolled following fulfillment of the following inclusion criteria: diagnosis of SCA confirmed by haemoglobin electrophoresis; age of 5 to 11 years; stable state; clinic attendance for at least 6 months prior to the study; and primary school attendance within Enugu metropolis. Children who had a history and urine sugar level suggestive of diabetes mellitus; a history suggestive of urinary tract infection; a personal history of epilepsy and; a history of diuretic medication were excluded.

The Controls were recruited from the primary schools attended by the corresponding Subjects following the informed consent of school authorities and the parents/caregivers. The haemoglobin genotypes of the pupils selected consecutively from the class register were determined to confirm their haemoglobin AA status. On the whole, 70 Subjects with haemoglobin SS and 70 Controls with haemoglobin AA were enrolled for the study.

### Data collection

A 26-item proforma consisting of open-ended questions on demographic data (such as the child’s age, parents’ educational attainment and occupation, as well as family size) and closed-ended questions on historical evaluation of enuresis based on the DSM-IV criterion (including family history of enuresis) was administered by the principal investigator (CIE) to the parents/caregivers of the enrolled Subjects and their class-mate Controls. Another 15-item proforma comprising open-ended questions on bio-data (child’s age and gender) and closed-ended questions on past medical history and evaluation for enuresis was also administered to the Subjects and Controls.

Social classification of the Subjects and the Controls was determined using the occupation and educational attainment of their parents or caregivers based on the method described by Oyedeji. [17]. Class I represents the upper social class, Classes II and III the middle class and Classes IV and V the lower class.

### Data analysis

Data entry and analysis were conducted using the Statistical Package for Social Sciences (SPSS) version 17.0 software. Descriptive statistics, including frequencies and percentages were used to summarize the socio-demographic data. Continuous variables were presented as means and standard deviation. Means were compared between continuous variables using 2 tailed Student’s *t*-test. The significance of the difference between the frequencies of categorical variables was ascertained using the Chi square test, or Fisher’s Exact Test as appropriate . Level of significance was at the 95 % confidence interval and *p* values less than 0.05. Data was subjected to multivariate analysis using logistic regression model with nocturnal enuresis as the dependent variable and the possible risk factors as the independent variables.

## Results

### Demographics

A total of 140 children were studied; 70 with SCA (Subjects) and 70 with haemoglobin AA genotype (Controls). The gender distribution showed 39 (55.7 %) males and 31 (44.3 %) females giving a male–female ratio of 1.3:1. The mean age of the Subjects and Controls with the same age range of 5 to 11 years was 8.37 ± 2.02 years (8.10 ± 1.96 years for males and 8.71 ± 2.05 years for females in both cohorts). The difference in their mean ages was not statistically significant (*t* = 1.78, *p* = 0.07).

### Prevalence of enuresis

As shown in Table 1, 23 (32.9 %) Subjects and 18 (25.7 %) Controls had enuresis although the difference was not statistically significant [OR (95 % CI) =1.41 (0.68–2.94), *x*^2^ = 0.86, *p* = 0.353]. However, 22 (31.4 %) Subjects and 15 (21.4 %) Controls had nocturnal enuresis. Only 1 (1.4 %) Subject and 3 (4.3 %) Controls had day-time enuresis. Notably, the difference in the prevalence of nocturnal enuresis in both cohorts was not statistically significant (*x*^2^ = 1.80, *p* = 0.180). The prevalence of day time enuresis was 1.4 % in Subjects and 4.3 % in Controls.

**Table 1:** Prevalence of nocturnal enuresis in subjects and controls

Nocturnal enuresis	Subjects (SS)	Controls (AA)	*χ* ^2^	*p* value
Yes	22 (31.4)	15 (21.4)	1.800	0.180
No	48 (68.6)	55 (78.6)		
Total	70 (100.0)	70 (100.0)		

### Prevalence of nocturnal enuresis versus risk factors

Eleven (29.7 %) Subjects in early school age (5–8 years) and 11 (33.3 %) in late school age (9–11 years) had nocturnal enuresis compared with 10 (27.0 %) and 5 (15.2 %) of the Controls in early and late school ages respectively. The difference between the prevalence of nocturnal enuresis in early versus late school age among the Subjects and the Controls was not statistically significant (*x*^2^ = 0.11, *p* = 0.746 for Subjects and *x*^2^ = 1.46, *p* = 0.227 for Controls). With respect to gender, 19 (48.7 %) male and 3 (9.7 %) female Subjects compared with 9 (23.1 %) male and 6 (19.4 %) female Controls had nocturnal enuresis (Table 2). Remarkably, the prevalence of nocturnal enuresis was significantly higher in male Subjects compared to female Subjects [OR (95 % CI) = 8.14 (2.12– 31.24), *x*^2^ = 12.21, *p* < 0.001]

**Table 2:** Prevalence of nocturnal enuresis in relation to gender

Nocturnal enuresis	Sex		OR	*p* value	95 % C.I for OR
	Male	Female			
Subjects (SS)					
Yes	19 (48.7)	3 (9.7)	8.143	0.002	2.122–31.240
No	20 (51.3)	28 (90.3)			
Total	39 (100.0)	31 (100.0)			
Controls (AA)					
Yes	9 (23.1)	6 (19.4)	1.161	0.801	0.363–3.713
No	30 (76.9)	25 (80.6)			
Total	39 (100.0)	31 (100.0)			

In Table 3, the distribution of the frequency of nocturnal enuresis among Subjects and Controls according to socio-economic class shows that 21, 27 and 22 Subjects and 15, 27 and 28 Controls belonged to the lower, middle and upper socio-economic classes (SEC) respectively. There was no significant difference between the distribution of socio-economic classes of Subjects and Controls (*x*^2^ = 1.72, *p* = 0.423). There was also no statistically significant relationship between socio-economic status and the prevalence of nocturnal enuresis among the Subjects (*p* = 0.962) and the Controls (*p* = 0.986).

**Table 3:** Relationship between nocturnal enuresis and socio-economic (SEC) status

Nocturnal enuresis	SEC class			*χ* ^2^	*p* value
	Lower	Middle	Upper		
Subjects (SS)					
Yes	7 (33.3)	8 (29.6)	7 (31.8)	0.077	0.962
No	14 (66.7)	19 (70.4)	15 (68.2)		
Total	21 (100.0)	27 (100.0)	22 (100.0)		
Controls (AA)					
Yes	3 (20.0)	6 (22.2)	6 (21.4)	0.028	0.986
No	12 (80.0)	21 (77.8)	22 (78.6)		
Total	15 (100.0)	27 (100.0)	28 (100.0)		

Furthermore, 12 (17.1 %) Subjects and 17 (24.3 %) Controls had parents with childhood history of enuresis but while 75 % of these 12 subjects had nocturnal enuresis themselves only 41.2 % of the 17 controls had nocturnal enuresis. Between the Subjects and the Controls, there was no significant difference in the prevalence of nocturnal enuresis among those with a positive history of enuresis in the parents (75.0 % versus 41.2 %; *p* = 0.071) or among those with a negative history (22.4 % versus 15.1 %; *p* = 0.325). However, among the Subjects, the prevalence of enuresis was significantly higher in those whose parents had a positive history of enuresis in childhood [OR (95 % CI) =10.39 (2.45–44.05), *p* = 0.002] (Table 4).

**Table 4:** Relationship between the prevalence of nocturnal enuresis and history of enuresis in the parents of subjects and controls

Parents’ history of childhood enuresis	Subjects		Controls		
	*N*	*n* (%)	*N*	*n* (%)	*χ* ^2^/*p*
Yes	12	9 (75.0)	17	7 (41.2)	3.25/0.071
No	58	13 (22.4)	53	8 (15.1)	0.97/0.325
*p* (Fisher Exact Test)		0.002		0.060	

*N* number studied, *n (%)* number (percentage) with enuresis

In the relationship between the prevalence of nocturnal enuresis and sibling history of enuresis in the Subjects and the Controls, there was no significant difference in enuretic versus non-enuretic Subjects (*x*2 = 2.85, *p* = 0.091) and enuretic versus non-enuretic Controls (Fisher Exact Test, *p* = 0.347) (Table 5). Eleven Subjects (29.7 %) in the early school age (5–8 years) and another 11 (33.3 %) in the late school age (9–11 years) had nocturnal enuresis. Ten (27.0 %) of Controls in the early school age and 5 (15.2 %) in the late school age had nocturnal enuresis (Table 6).

**Table 5:** Relationship between the prevalence of nocturnal enuresis and history of enuresis in siblings of subjects and controls

Enuresis in siblings	Subjects		Controls			
	*N*	*n* (%)	*N*	*n* (%)	*χ* ^2^	*p*
Yes	25	11 (44.0)	19	6 (31.6)	0.70	0.402
No	45	11 (24.4)	51	9 (17.6)	0.67	0.413
*χ*2*/p*		2.85/0.091		NA/0.347^a^		

*N* number studied, *n (%)* number (percentage) with enuresis

^a^Fisher Exact Test

**Table 6:** Prevalence of nocturnal enuresis in relation to age

Nocturnal enuresis	Age (years)		*χ* ^2^	*p* value
	5–8	9–11		
Subjects (SS)				
Yes	11 (29.7)	11 (33.3)	0.105	0.746
No	26 (70.3)	22 (66.7)		
Total	37 (100.0)	33 (100.0)		
Controls (AA)				
Yes	10 (27.0)	5 (15.2)	1.461	0.227
No	27 (73.0)	28 (84.4)		
Total	37 (100.0)	33 (100.0)		

### Predictors of nocturnal enuresis

In Table 7, male gender [OR (95 % CI) = 20.18 (3.313–122.951), *p* = 0.001], age group 5–8 years [OR (95 % CI) = 0.23 (0.056–0.967), *p* = 0.045], and parental history of nocturnal enuresis [OR (95 % CI) = 20.47 (2.690–155.704), *p* = 0.004] were predictors of nocturnal enuresis in the Subjects. However, none of these factors were predictors of nocturnal enuresis in the Controls (Table 8).

**Table 7:** Multivariate analysis of predictors of nocturnal enuresis in subjects

	*p* value	OR	95 % C.I. for OR	
			Lower	Upper
Sex (male)	0.001	21.126	3.452	129.282
Age group (5–8 years)	0.046	0.233	0.056	0.973
Social Class (upper)	0.670	0.714	0.151	3.375
SIBNE	0.233	2.321	0.582	9.257
PNE	0.003	22.005	2.929	165.327

*SIBNE* Sibling nocturnal enuresis, *PNE* Parental nocturnal enuresis

**Table 8:** Multivariate analysis of predictors of nocturnal enuresis in controls

	*p* value	OR	95 % C.I. for OR	
			Lower	Upper
Sex (male)	0.816	0.855	0.229	3.197
Age group (5–8 years)	0.158	2.625	0.687	10.026
Social Class (upper)	0.660	1.353	0.352	5.197
SIBNE	0.107	3.016	0.787	11.553
PNE	0.018	5.216	1.327	20.503

*SIBNE* Sibling nocturnal enuresis, *PNE* Parental nocturnal enuresis

## Discussion

### Prevalence of nocturnal enuresis

In the current study, school-aged children with SCA showed a tendency for nocturnal enuresis more than their non-affected class-mates with normal haemoglobin genotype. Furthermore, the prevalence of nocturnal enuresis was significantly higher among the male children with SCA than in their male controls. It was also higher among the subjects with parental history of nocturnal enuresis in childhood than in controls whose parents had a similar history. In comparison, previous studies indicate that children with SCA have a significantly higher prevalence of nocturnal enuresis than those with normal haemoglobin genotype [9–14]. For instance, the study in South-west Nigeria reported prevalence values of 41.6 % in children with SCA and 18.5 % in those with normal haemoglobin [12]. Another study in North-west Nigeria also documented a prevalence rate of 47.1 % in children with homozygous sickle-cell disease, which was significantly higher than that obtained in controls [13]. Similarly, Jordan et al. [13] reported that life time rates of nocturnal enuresis among children with the disease exceeded population prevalence and sibling control rates. Other workers noted that 35.7 % of children with the disease met the DSM-IV criteria for nocturnal enuresis compared to 5 % prevalence in normal children [14]. Elsewhere in Congo Brazzaville, other investigators documented a prevalence of 50.9 % in SCA subjects and 16.4 % in those with normal haemoglobin [9]. Interestingly, the reported prevalence values in children with SCA from these studies [9, 12–14] are higher than the prevalence of 31.4 % in the present study.. The relatively high prevalence figures of these previous studies may be attributed to differences in definition of enuresis [9], and subject selection bias [12]. Whereas the age range of the subjects in our study was 5–11 years, the study in South-west Nigeria used subjects aged between 2 and 20 years [12]. It is likely that some of the subjects who might not have achieved bladder control were wrongly labeled to have enuresis; thus accounting for its higher prevalence. In addition, confounding factors such as diabetes mellitus, epilepsy and urinary tract infection which can also cause nocturnal enuresis were not excluded in these studies [9, 12].

Furthermore, the prevalence of nocturnal enuresis among the controls in the current study is higher than the values noted in previous studies [9, 12–14]. The reason for the comparatively lower prevalence of nocturnal enuresis among the controls in these studies (which enrolled children beyond the school-age bracket) may be the observed decrease in the prevalence of enuresis with age, particularly after the age of sixteen years and among children with normal haemoglobin [9, 12, 18–20]. This phenomenon was attributed to spontaneous improvement by Murat and co-workers [19].

### Risk factors for nocturnal enuresis

The male predominance of nocturnal enuresis among children with SCA noted in our study is in tandem with the findings from other studies [10, 12, 13, 21]. A similar trend has also been reported among children with normal haemoglobin genotype [13, 22–24]. This suggests that factors contributing to enuresis in normal children such as slower maturation and reduced responsiveness to toilet training in boys [23], as well as more frequent developmental delays [24], are equally important in children with SCA. In contrast, other researchers have documented a female predominance in their subjects [9]. Generally, limited progress has been made in establishing the etiology and pathophysiology of nocturnal enuresis in subjects with the disease. One author had noted that nocturnal enuresis in children can result from an interaction of detrusor instability, delayed arousal from sleep and nocturnal polyuria [25]. In addition, Kawauchi et al. [26] observed that in enuretic children, the bladder capacity during sleep was significantly smaller than the day-time functional capacity; underscoring the role of the inability to hold urine during sleep as an important cause of nocturnal enuresis. Nonetheless, in a recent review by Wolf et al. [27], the postulated non-mutually exclusive causes of nocturnal enuresis in subjects with sickle-cell disease were listed as hyposthenuria leading to nocturnal polyuria, decreased bladder capacity or nocturnal bladder over activity, increased arousal thresholds, and sleep-disordered breathing (SDB).

Another risk factor for nocturnal enuresis observed in children with SCA in the present study is the history of parental enuresis during childhood which contrasts with the report in one previous study [13]. However, sibling history of enuresis was not a significant risk factor in our study. This finding further underscores the fact that while genetic factors may play a role in determining the prevalence of enuresis among parents and their off-springs, somatic and psychosocial factors may modulate these genetic factors in different off-springs of the same parents [28]. The effect of these modulatory influences is corroborated by Jordan et al. [11], who observed a higher prevalence of nocturnal enuresis among children with SCA compared to their sibling controls. Better still, the higher prevalence of enuresis among subjects with parental rather than sibling history of NE, in contrast to the controls may further suggest that these somatic and psychosocial factors have less modulatory influence on the genetic factors in children with SCA than in non-affected children. We observed that 75 % of the 12 SCA children with parental history of NE had personal history of NE while only 41.2 % of the 17 controls with parental history of NE had similar personal history. This suggests that parental history of NE may play a more significant role in children with SCA than in those with normal haemoglobin.

The present study has also noted that socio-economic status did not have any significant impact on the prevalence of nocturnal enuresis; which compares with the reports by Ogunrinde et al. in Nigeria [13], Readett and co-workers in Jamaica [21], as well as Hansakunachaj et al. in Thailand [29]. This finding is however at variance with previous observations [22–24], which noted that enuresis was more frequent among normal children from the lower socio-economic class. Conversely, a study in mid-west Nigeria reported a higher prevalence of nocturnal enuresis among normal children from higher socio-economic class [30]. Thus, there appears to be no unanimity on the role of socio-economic status as a risk factor for nocturnal enuresis among affected and unaffected children. It may well reflect the improved awareness of health and health-related matters across the social classes. Readett and co-workers attributed this finding to the relative unreliability of social amenities in predicting social status [21].

### Study limitation

The power of recall by some parents/care-givers might have been be a source of bias in the prevalence of nocturnal enuresis among the study cohorts. Again, this memory lapse might have affected our evaluation of parental history of nocturnal enuresis in childhood as a potential socio-demographic risk factor.

## Conclusions

In conclusion, children with SCA have a tendency to develop nocturnal enuresis when compared to their counterparts with normal haemoglobin genotype. In addition, gender and parental history of childhood nocturnal enuresis are potential socio-demographic risk factors. It is hoped that the findings of this study will provide base-line reference data for future studies in this clime. It is recommended that school-aged children with SCA who are in steady state should be frequently evaluated for nocturnal enuresis, particularly if they are male children and have parents with history of nocturnal enuresis in childhood.

## Abbreviations

DSM-IV: Diagnostic and Statistical manual of Mental disorders-4^th^ edition
NE: Nocturnal enuresis
SCA: Sickle-cell anaemia
SDB: Sleep disordered breathing
SEC: Socio-economic class
